# Supplementary material for: Progerin Expression Induces Inflammation, Oxidative Stress and Senescence in Human Coronary Endothelial Cells
Source: Cells. 2020 May 12;9(5):1201. doi: 10.3390/cells9051201 (PMC7290406; doi:10.3390/cells9051201)
Supplement: Supplementary file 1 [file cells-09-01201-s001.pdf]

cells

MDPI

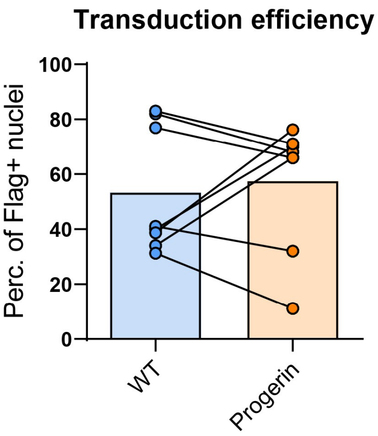

Supplemental Figure S1. Transduction efficiency assessed by the number of Flag-positive cells. Early-confluent HCAECs were transduced with Flag-tagged recombinant adenovirus containing WT-prelamin A (WT) or progerin (Prog) for 72 h. The transduction efficiency was determined by the percentage of Flag-positive cells as assessed by fluorescence microscopy. Values for each experiment (represented by symbols linked by a line) are presented. Comparison of the transduction efficiency between experiments found no difference (p = 0.6424 for paired Student t-test analysis).

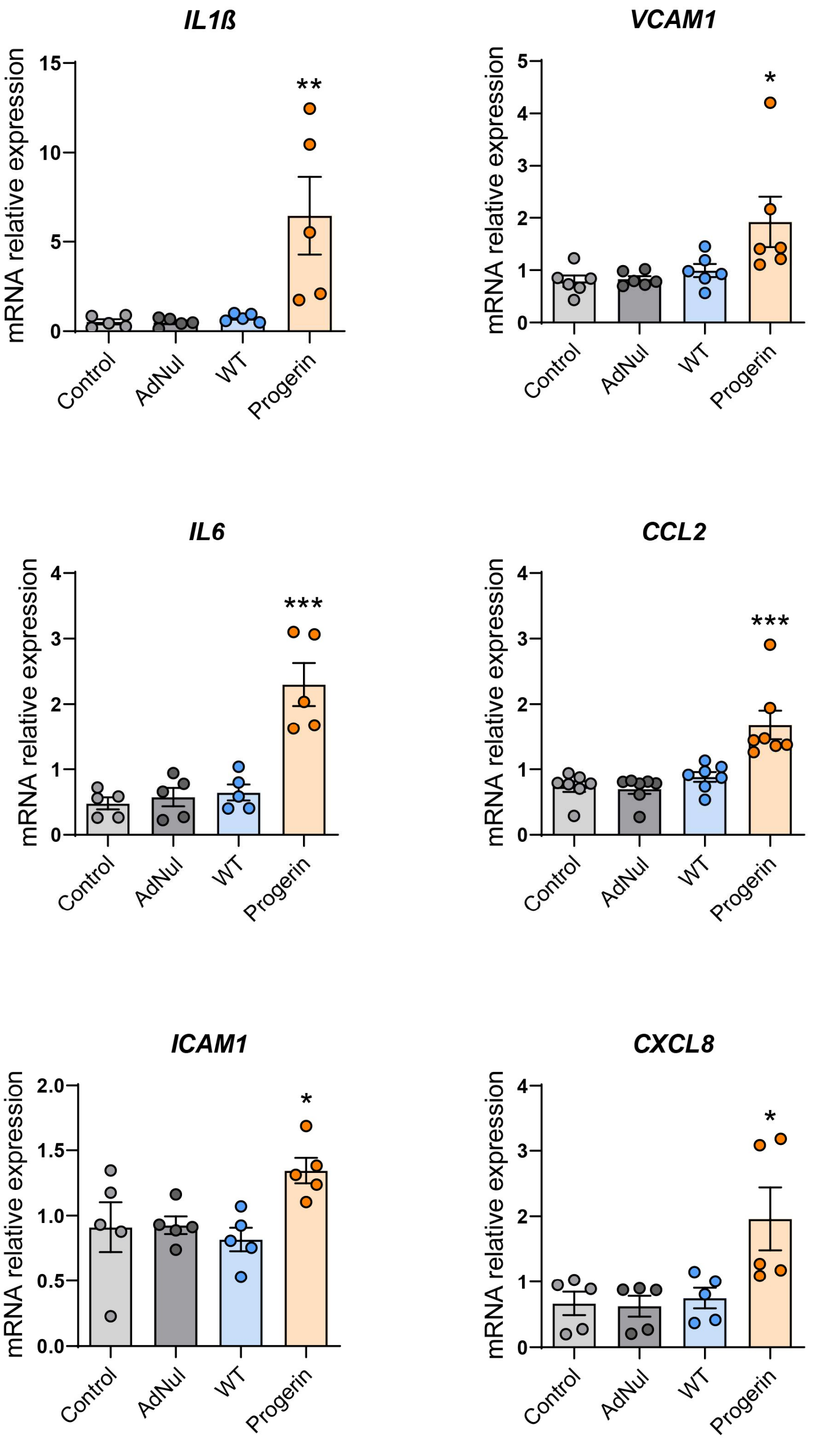

Supplemental Figure S2. mRNA expression of proinflammatory markers and cell adhesion molecules in HCAECs. Early-confluent HCAECs were transduced or not (Control) with Flag-tagged recombinant adenovirus containing WT-prelamin A (WT) or progerin for 72 h or with an empty vector (AdNul). Relative mRNA expression is expressed as mean ± SEM. Statistical difference was determined using ANOVA followed by a Dunnett post hoc test. * $p < 0.05$, ** $p < 0.01$, *** $p < 0.001$ vs. WT.

cells

MDPI

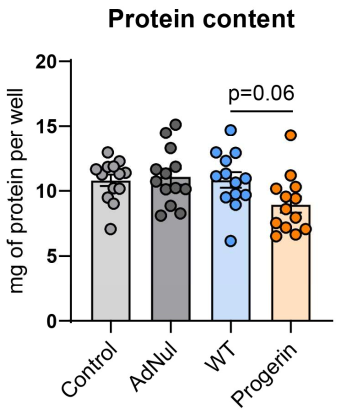

Supplemental Figure S3. Protein content per well (6-wells plate) in control and transfected HCAECs in all comparable experiments. Early-confluent HCAECs were transduced or not (Control) with Flag-tagged recombinant adenovirus containing WT-prelamin A (WT) or progerin for 72 h or with an empty vector (AdNul). Data are expressed as mean ± SEM. Statistical difference was determined using ANOVA followed by a Dunnett post hoc test. There was no differences in each well protein content under the different conditions compared to WT although a trend towards a decreased protein content was observed upon progerin expression.
